# Urban Flooding Prediction Method Based on the Combination of LSTM Neural Network and Numerical Model

**DOI:** 10.3390/ijerph20021043

**Published:** 2023-01-06

**Authors:** Jian Chen, Yaowei Li, Changhui Zhang, Yangyang Tian, Zhikai Guo

**Affiliations:** Water Conservancy College, North China University of Water Resources and Electric Power, Zhengzhou 450046, China

**Keywords:** urban flooding, LSTM neural network, numerical model of flooding, quickly predict

## Abstract

At present, urban flood risk analysis and forecasting and early warning mainly use numerical models for simulation and analysis, which are more accurate and can reflect urban flood risk well. However, the calculation speed of numerical models is slow and it is difficult to meet the needs of daily flood control and emergency. How to use artificial intelligence technology to quickly predict urban flooding is a key concern and a problem that needs to be solved. Therefore, this paper combines a numerical model with good computational accuracy and an LSTM artificial neural network model with high computational efficiency to propose a new method for fast prediction of urban flooding risk. The method uses the simulation results of the numerical model of urban flooding as the data driver to construct the LSTM neural network prediction model of each waterlogging point. The results show that the method has a high prediction accuracy and fast calculation speed, which can meet the needs of daily flood control and emergency response, and provides a new idea for the application of artificial intelligence technology in the direction of flood prevention and mitigation.

## 1. Introduction

The increased rate of urban development, the increase in the number of less permeable pavements, and the increased probability of extreme rainfall conditions due to global warming have led to frequent urban flooding [1,2]. For example, in 2012 “7.21”, Beijing’s extraordinarily heavy rainfall caused 1.602 million people to be affected and economic losses amounted to RMB 11.64 billion. Additionally, in 2014 “7.27”, Hefei’s heavy rainfall event caused nearly one million people to be affected and economic losses amounted to RMB 1.2 billion. In July 2021, 14,531,600 people in the province were affected by the extraordinarily heavy rainfall in Zhengzhou, with a direct economic loss of 114.269 billion yuan [3]. Urban flooding poses a huge threat to the safety of residents’ lives and property, and has become one of the serious illnesses affecting the healthy development of cities. Early foreknowledge, prediction and prevention of urban flooding risks, as well as the timely release of early warning information to guide citizens’ lives and protect people’s lives and properties, are urgent issues in urban flooding risk management.

Scholars have conducted in-depth studies on the coupling mechanisms of surface models, river models and underground pipe network models from the perspective of hydrology and hydrodynamics, and have achieved fruitful research results [4,5]. Huang Guoru et al. [6] proposed the coupled hydrology and hydrodynamics model IHUM (Integrated Hydrology and Hydrodynamics Urban Flood Model), which couples a one-dimensional SWMM model with a two-dimensional hydrodynamic model. Liu Jiahong and Mei Chao et al. [7,8] proposed coupling the tract hydrological model with the drainage system hydrodynamic model through a rain grate. Zang Wenbin [9] investigated the model mechanism nested simulation method in terms of both hydrology and hydrodynamics, and the coupling mechanism between the models in terms of surface drainage and surface river connection. All of these methods use the outlet flow process of the catchment area calculated based on the hydrological method as the inlet condition of the drainage network, realizing a direct dynamic two-way coupling between the distributed hydrological model and the hydrodynamic model, which can balance the accuracy of the model and the efficiency of the calculation. Xu Zongxue et al. [10] considered the hydrological and hydrodynamic models as a whole and solved the control equations jointly.

However, with the development of cities, the coupling of surface, river and pipe networks is becoming more and more complex, the grid size of the model is getting smaller and smaller, the grid volume is huge, and the very complex calculations required for the huge grid volume consume huge computational resources. Although hardware such as supercomputers and GPU parallel computing has emerged in recent years [11,12] and the speed of numerical simulation has improved, we still cannot meet the demand of urban flood control and emergency response time. Therefore, it is necessary to find a new method that can quickly analyze the risk of urban flooding under current storm conditions, predict the risk of storm flooding in advance, and dispatch personnel and materials in time to reduce the risk of flooding.

In recent decades, Artificial Intelligence (AI) technology has made great strides in the fields of computer vision, natural language processing, machine translation, robotics and control, and bioinformatics [13]. At present, AI has been successfully applied to assist decision-making in weather forecasting and the identification of some extreme disasters [14,15], and is also widely used in “smart water” applications such as water resource management and scheduling of water supply and drainage plants in many cities [16]. Convolutional Neural Network Convolutional Neural Networks (CNN) were initially used in the recognition of flood evolution and road waters [17,18], but the algorithm requires high computer computing power and training samples, making it difficult to promote this application. Artificial neural network technology is inseparable from the training of a large number of high-quality data samples, and in practice, both the number of rainfall fields and the measured waterlogging data are far from meeting the demand for samples in terms of quality and quantity, which limits the effective application of artificial neural network technology in urban flood risk prediction. The problem of how to introduce artificial neural network technology into the storm-flooding scenario, where the number of training samples is small and the annotated data are small is number, and how to quickly predict the flooding of the lower cushion surface with a small number of storm samples is a major concern and needs to be addressed. On the other hand, there are many different algorithms for artificial neural network models, and it is also a problem to be explored and researched to select the algorithm with a fast operation speed and high prediction accuracy for rapid prediction of storm water flooding.

The Long Short-Term Memory (LSTM) neural network model is a relatively mature and widely used artificial neural network model at present. This paper combined the LSTM neural network model and hydrological hydrodynamics model to propose a new method for urban flooding risk prediction, and used the method to simulate and predict the waterlogging process at each waterlogging point in the central city of Zhoukou. The results show that the prediction results of the method are less inaccurate compared with the measured waterlogging monitoring data and numerical simulation results, and the prediction accuracy is high. Moreover, the calculation speed is fast, which greatly shortens the prediction time of waterlogging risk and effectively solves the problem of timeliness in urban waterlogging prediction and warning.

## 2. Materials and Methods

### 2.1. Research Process

**Figure 1:**
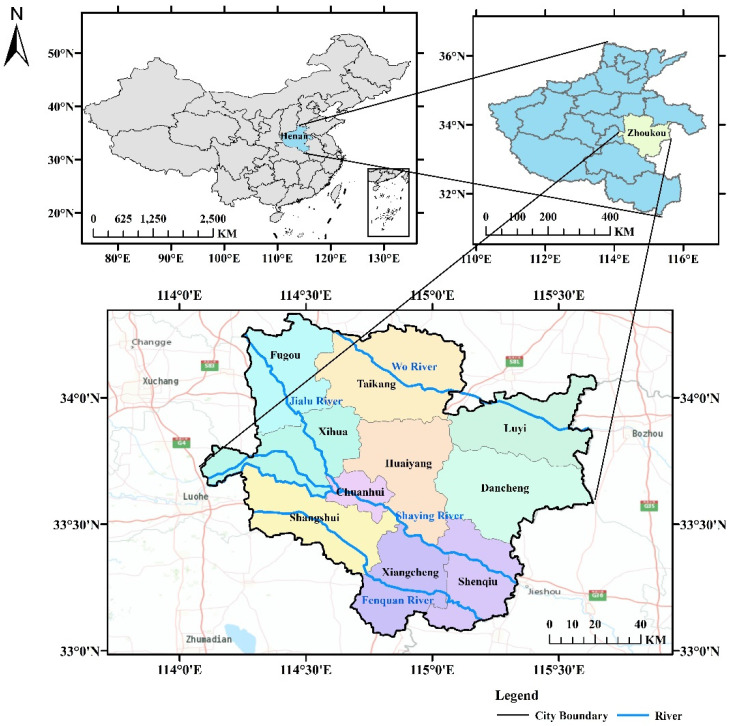
Zhoukou city location map.

**Figure 2:**
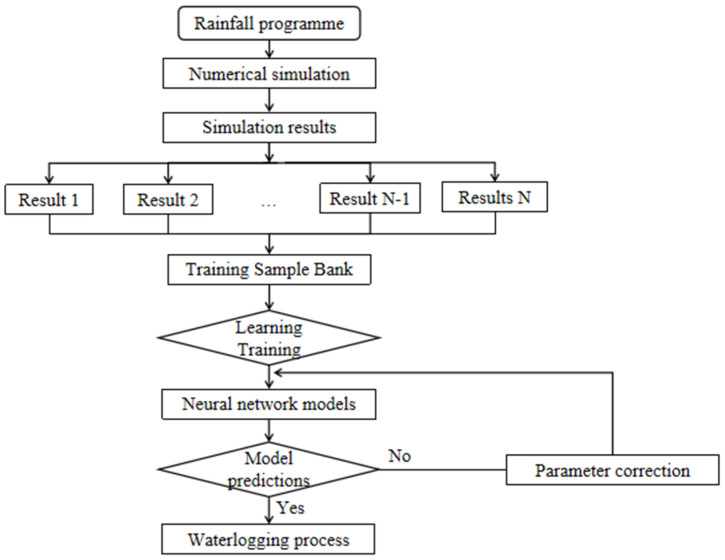
Technical flow chart.

Zhoukou is located in the East Henan Plain, in the southeastern part of Henan Province. The location map of Zhoukou City is shown in Figure 1. An analysis of the current urban built-up land in Zhoukou shows that the proportion of hardened underlayment is high and the indicators for green space and square land are significantly low. Rainfall in Zhoukou is mostly in the form of heavy rainfall. The fast flow rate of rainfall patterns can cause high peak flow rates of rainfall runoff, resulting in high pressure on the drainage system, which can easily cause waterlogging in the city. Therefore, in this paper, a numerical model was built to simulate the rainfall scenario with a 3 h short calendar period intense rainfall process in the central city of Zhoukou, and the simulation results were used as the data driver for the neural network prediction at each waterlogging point. Finally, the trained neural network model was used to predict the waterlogging process at each waterlogging point using the storm-flooding samples that did not participate in the training as the prediction samples, in order to test the prediction effect of the neural network model. The specific technical process is shown in Figure 2.

### 2.2. Rainfall Programme

The rainfall scenarios include actual short historical intense rainfall processes from 1975–2019 and designed rainfall based on the Zhoukou City storm intensity formula. The greater the amount of data in the training sample pool, the more accurate the neural network model. Therefore, the rainfall scenarios were used as input conditions for the numerical model, and the rainfall-inundation samples for a variety of rainfall conditions were obtained through numerical model simulations. The rainfall-flooding samples were used to train the neural network model for learning. A total of 832 rainfall scenarios of 3 h duration were calculated, resulting in 20,422 storm-flooding samples. The number and type of samples obtained met the requirements of the neural network model in terms of the number and type of samples learnt. For training, 90% of the samples were selected for training and 10% for testing. The trained neural network model can thus predict the waterlogging process at the waterlogging point under various rainfall conditions.

### 2.3. Urban Flooding Simulation Model

#### 2.3.1. One-Dimensional River Hydrodynamic Model

The one-dimensional river hydrodynamic model will simulate the evolution of floods within the main and tributary streams by generalizing the river channels within the study area. According to the model requirements, river centerlines and river cross-sections within the central city were extracted, river networks were established and river crossings were identified. The 1D river model constructed includes 12 rivers with 682 cross sections.

#### 2.3.2. 2D Surface Diffuse Flow Model

The construction of the 2D surface diffuse flow model consists mainly of the construction of the base terrain and the processing of the subsurface data. The dem terrain data were processed and converted to the ASCII text format mainly by Arcgis. Land use data such as buildings, roads and green areas were superimposed on the terrain. The area simulated by the 2D surface diffuse flow model in the central city of Zhoukou is about 145.75 km^2^, using a rectangular grid with a grid size of 2 m × 2 m; the number of grids is 3.64 × 10^7^ pcs with a grid size of 4 m^2^.

#### 2.3.3. 1-Dimensional Stormwater Pipe Network Model

The 1D stormwater pipe network model mainly includes rainfall runoff simulation and pipe network hydraulics simulation. The actual stormwater network was generalized into a pipe network model through Arcgis and the relevant properties of the inspection wells and pipes were checked and improved through data topology. The network model consists of 11,029 inspection wells and 11 pumping stations.

### 2.4. Training Sample Generation

**Figure 3:**
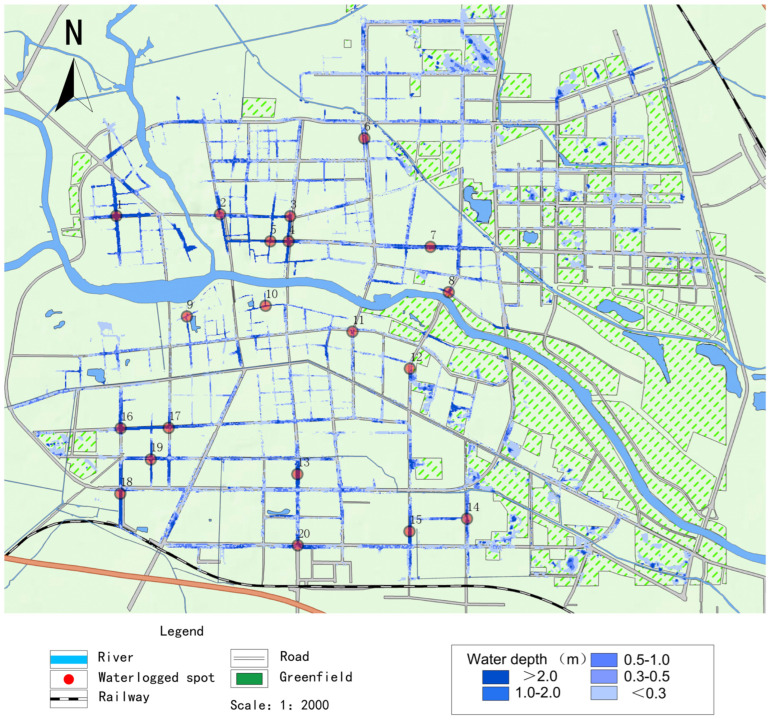
2021.7.20 Distribution of major waterlogged sites.

Before training the neural network, accurate training samples need to be obtained. Therefore, the numerical model was validated using actual rainfall processes to make the rainfall-flooding samples more accurate. After validation, the simulation results of the numerical model have small errors compared with the actual measured values, which can meet the requirements in terms of the quality of the learning samples. This paper takes the period of 2021.7.20 in Zhoukou City as an example, choosing 20 major waterlogging points in the central city, and the distribution of waterlogging points is shown in Figure 3. The measured maximum ponding depth data were compared with the maximum ponding depth simulated by the numerical model, as shown in Table 1. It can be seen that the simulated and measured values are close to each other, with the difference being within 5 cm, meeting the requirements of the neural network model in terms of the samples’ quality for learning.

This paper used this numerical model to carry out simulations of the actual short historical heavy rainfall processes that occurred from 1975–2019 and the designed rainfall based on the storm intensity formula for Zhoukou City, and the resulting rainfall-inundation samples were used as the data driver for the neural network model to construct an artificial neural network model for each major waterlogging point.

### 2.5. LSTM Neural Network Model

**Figure 4:**
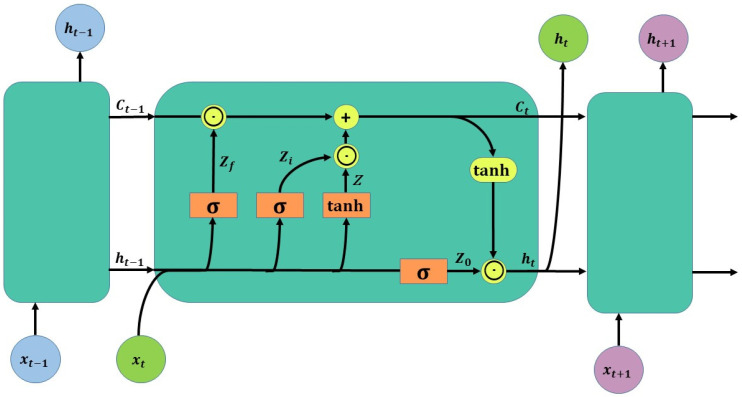
Structure of LSTM.

The Long Short-Term Memory (LSTM) neural network [19] is a modified model of the Recurrent Neural Network Model (RNN), which introduces self-looping on top of the RNN neural network, allowing the model to capture long-range dependencies and non-linear information. The long and short-term memory neural network consists of an input layer, one or more hidden layers and an output layer, where the hidden layer introduces “input gates”, “forget gates” and “output gates” to control the flow of information. The structure of the LSTM is shown in Figure 4.

First, *X_t_* and the *h_t_*_−1_ passed down from the previous state are used as the current input, and the two are trained by LSTM splicing to obtain four states.

(1) $$Z=\tanh(WX_{t}+Wh_{t-1})$$

(2) $$Z_{i}=(W_{i}X_{t}+W_{i}h_{t-1})$$

(3) $$Z_{f}=(W_{f}X_{t}+W_{f}h_{t-1})$$

(4) $$Z_{0}=(W_{0}X_{t}+W_{0}h_{t-1})$$

where *W* is the weighting matrix. *Z_f_*, *Z_i_*, and *Z*_0_ are multiplied by the splice vector in the weight matrix and then converted to a value between 0 and 1 by the activation function sigmoid to act as a gating state. We took *z* as input data and used the tanh activation function to convert it to a value between 1 and −1. The two activation functions are calculated as follows.

(5) $$\tanh(x)=\frac{e^{x}-e^{-x}}{e^{x}+e^{-x}}$$

(6) $$\sigma (x)=\frac{1}{1+e^{-x}}$$

The next four states are calculated as follows

(7) $$C_{t}=Z_{f}⊙C_{t-1}+Z_{i}⊙Z$$

(8) $$h_{t}=Z_{0}⊙\tan h(C_{t})\;$$

where ⊙ is the Hadamard Product, i.e., the corresponding element of the operation matrix is multiplied. ⊕ represents the matrix addition. The computed *Z_f_* (f for forget) is the forgotten gating, and the selected gating signal is controlled by *Z_i_* (i for information); and the results of the above two steps are summed to obtain the *C_t_* transmitted to the next state. Compared to traditional recurrent neural networks, the long and short-term memory network model has selective memory and interaction within the time series by gating states, which is ideal for solving the non-stationary nature of the precipitation time series, as well as its stochastic nature.

### 2.6. Waterlogging Model Prediction Construction

The LSTM neural network model was developed for each of the 20 major waterlogging points in Zhoukou city center in Figure 3. The water depth at each point is mainly related to the rainfall and water depth of the previous time series at the point. The input variables of this neural network model are six quantities, namely the rainfall in the previous three time series {R_t−3_, R_t−2_, R_t−1_} and the water depth in the previous three time series {H_t−3_, H_t−2_, H_t−1_} of the water accumulation point, and the output variable is one quantity, the water depth H_t_ in the next one time series. The neural network model was continuously trained to predict the depth of ponding over time throughout the rainfall process.

For training, 90% of the storm-flooding samples were selected as training samples and the remaining 10% were used as test samples, respectively. The trained model was also used to predict the waterlogging for the storm process that did not participate in the model training. Finally, the degree of similarity between the two curves was determined by calculating the coefficient of determination R² between the predicted and simulated data. The closer the coefficient of determination R² is to 1, the better the fit of the two curves is, i.e., the closer the prediction result of the neural network model is to that of the numerical model.

## 3. Results and Discussion

**Figure 5:**
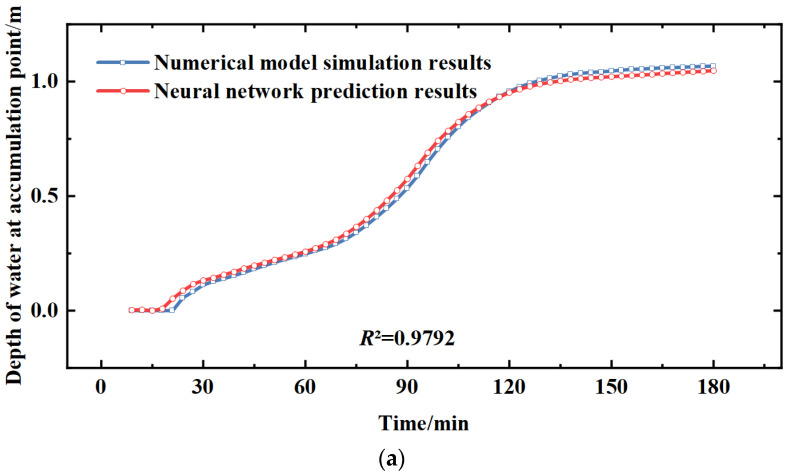

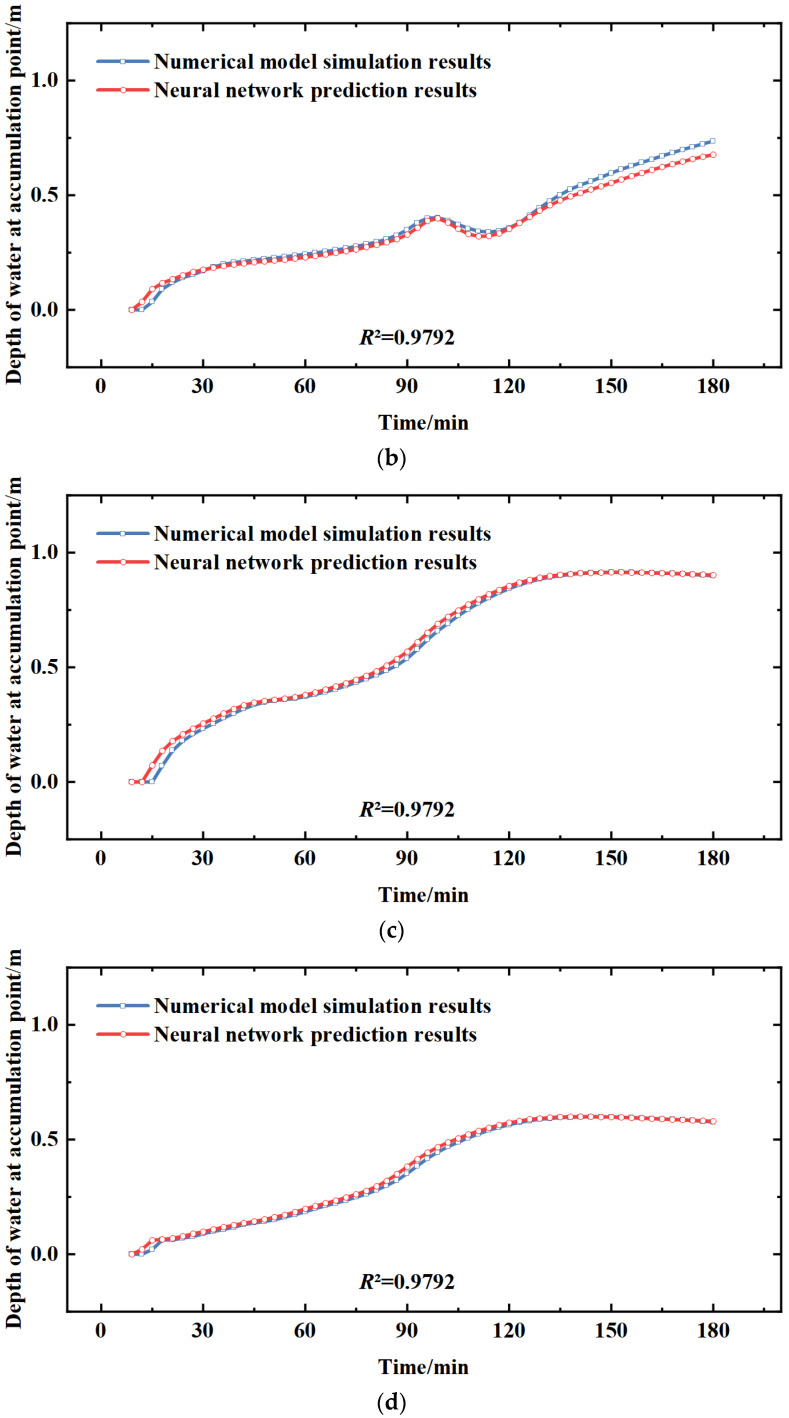

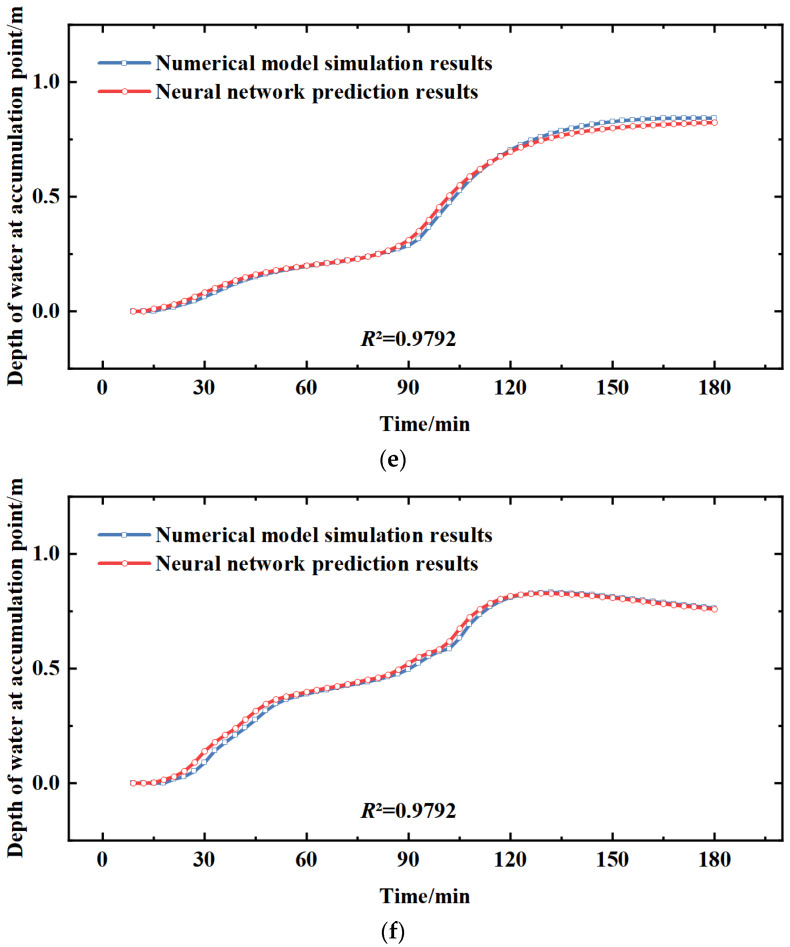
Comparison of simulation results of design rainfall scenarios based on the storm intensity formula. (**a**) Intersection of Construction Road and Yinzhu Road, (**b**) Bayi Road North, (**c**) Zhou Shi gate, (**d**) Area around Sanlian Hang, (**e**) Yellow River Road West, (**f**) Zhong Yuan Road South.

The trained neural network prediction model was used to predict the designed rainfall and the actual rainfall process based on the Zhoukou rainstorm intensity formula. For the calculation, the designed rainfall scenario was a Chicago rainfall pattern with general rainfall across the region. A 20-year design rainfall with r = 0.5 for the Chicago rainfall pattern was selected and the results are shown in Figure 5.

The area was divided into six different drainage areas and one ponding point from each drainage area was selected for analysis. A total of six ponding points were selected. It can be seen that the coefficients of determination R² are all above 0.95, and the error between the waterlogging process simulated by the numerical model and that predicted using the LSTM model is small. Figure 5 shows the designed rainfall using the equation based on the intensity of the storm in Zhoukou, with a simulation duration of 3 h. It can be seen that the designed rainfall r = 0.5, the rain front appears at the middle moment, and the ponding starts to gradually accelerate at each ponding point at the middle moment. The trend of water accumulation at the six ponding points, which are distributed in various drainage areas throughout the central city, is basically similar. It can be seen that the numerical model of urban flooding developed in this paper and the LSTM neural network model trained based on the results of the flooding model both reflect the relationship between the water depth at each ponding point and with the variation of rainfall intensity and time. The prediction results are close to the actual situation.

The design rainfall continuity calculated based on the storm intensity formula is good, but the continuity of the measured rainfall data is poor, so the maximum ponding depth calculated by the LSTM neural network model was selected for comparison with the actual monitored maximum ponding depth. The model was used to predict the maximum ponding depth results for each ponding point during 2021.7.20, where no training was involved. The results of the comparison with the actual measured maximum ponding depths are shown in Table 2.

As seen in Table 2, for the 20 major ponding points, the average error between the maximum ponding depth simulated by the numerical model and the measured values is 5.11%, while the average error between the maximum ponding depth simulated by the LSTM neural network model and the measured values is 6.34%. As the LSTM model in this paper is driven by using the results of numerical model simulations as input, there is an accumulation of errors through continuous training, and the error between the LSTM model and the measured values increases by 1.23% compared to the numerical model. However, for actual rainfall with poor continuity, the error between the LSTM model and the numerical model is only 2.53%.

The area of this study is approximately 100 km² and it would take several hours to calculate 3 h of rainfall using the numerical model, however, the same rainfall scenario would only take a few seconds to calculate using the LSTM neural network model. The computation speed of the neural network is significantly faster. The computational speed of the numerical model is closely related to the size of the computational range, the number of grids and the duration of the simulation, and cannot meet the needs of real-time simulations. The LSTM neural network model prediction method proposed in this paper is fast, with few errors, and can effectively meet the needs of emergency flood control.

## 4. Conclusions

In this paper, the LSTM neural network model was combined with the numerical model of urban flooding to propose a new method for rapid prediction of urban flooding accumulation. The results show that the artificial neural network model has obvious advantages in prediction, mainly in the following ways:

(1) The LSTM neural network simulation predicts the actual rainfall maximum water accumulation depth with an average error of 6.34%, which has a high prediction accuracy.

(2) The calculation speed of LSTM neural network model prediction is very fast, which can meet the demand of emergency flood control.

As this neural network model uses the calculated results of the numerical model as training samples, errors accumulate. With the increasing amount of measured rainfall flood data and the progressive development of the neural network model algorithms, the sample requirements have been reduced. A total of 832 rainfall scenarios with a duration of 3 h were calculated for this model, yielding 20,422 storm flood samples. For training, 90% of the samples were selected for training, and the error between the trained results and the numerical model was only 2.53%, with an average error of 5.11% between the measured values. The accuracy can be greatly improved if the measured storm flood data are used as the driver, allowing for quicker predictions and more accurate results.

## Figures and Tables

**Table 1:** 2021.7.20 Comparison of measured and numerically modeled ponding depths at inundated water points.

Serial Number	Location of Ponded Water	Measured Water Depth /cm	Simulated Water Depth /cm	Difference /cm
1	Bayi Road North	60	65	5
2	Junction of Qi Yi Road and Da Qing Road	40	42	2
3	Near Gongnong Road and Yuxin Street	55	58	3
4	Intersection of Construction Road and Yinzhu Road	30	31	1
5	Area around Sanlian Hang	80	76	−4
6	West Street Community	80	84	4
7	Jianxi Street area	60	56	6
8	Area near Anju Road	40	45	5
9	Fumin Road South area	40	43	3
10	Hanyang Road South area	40	41	1
11	Jiancai Road area	40	38	−2
12	Gao Zhuang Community	40	42	2
13	Wenchang road	60	58	−2
14	Bayi Road and Construction	60	63	3
15	Zhou Shi gate	40	42	2
16	Civil Service District A	60	63	3
17	Yuxin Street East	40	42	2
18	Tai Hao Road, Zijingcheng District	35	39	4
19	Junction of Chaoyang Road	30	34	4
20	Tai Hao Road, Wu Yi junction	30	36	6

**Table 2:** 2021.7.20 Comparison of measured and simulated water depths at inundated water accumulation points.

Serial Number	Location of Ponded Water	Measured Maximum Water Depth(cm)	Numerical Model Simulates Maximum Depth of Water Accumulation/cm	LSTM Model Simulates Maximum Depth of Water Accumulation/cm	Numerical Model and Measured Maximum Depth of Water Accumulation Error/%	LSTM Model and Measured Maximum Ponding Depth Errors/%	LSTM Model and Numerical Model Maximum Ponding Depth Error/%
1	Bayi Road North	60	65	66	8.33	10	1.54
2	Junction of Qi Yi Road and Da Qing Road	40	42	44	5	10	4.76
3	Near Gongnong Road and Yuxin Street	55	58	57	5.45	3.64	1.72
4	Intersection of Construction Road and Yinzhu Road	30	31	32	3.33	6.67	3.23
5	Area around Sanlian Hang	80	76	78	5	2.5	2.63
6	West Street Community	80	84	85	5	6.25	1.19
7	Jianxi Street area	60	56	57	6.67	5	1.79
8	Area near Anju Road	40	43	44	7.5	10	2.33
9	Fumin Road South area	40	43	42	7.5	5	2.33
10	Hanyang Road South area	40	41	43	2.5	7.5	4.88
11	Jiancai Road area	40	38	38	5	5	0
12	Gao Zhuang Community	40	42	38	5	7.5	2.38
13	Wenchang road	60	58	59	3.33	1.67	1.72
14	Bayi Road and Construction	60	63	62	5	3.33	1.59
15	Zhou Shi gate	40	42	43	5	7.5	2.38
16	Civil Service District A	60	63	64	5	6.67	1.59
17	Yuxin Street East	40	42	41	5	2.5	2.38
18	Tai Hao Road, Zijingcheng District	35	34	33	2.8	5.71	2.94
19	Junction of Chaoyang Road	30	32	33	6.67	10	3.13
20	Tai Hao Road, Wu Yi junction	30	31	32	3.33	6.67	3.23
	Average error				5.11	6.34	2.53

## Data Availability

Data and materials are available from the corresponding author upon request.
